# Hospital Meetings, &c.

**Published:** 1905-05-13

**Authors:** 


### HOSPITAL MEETINGS, &c.

### ST. MARY'S HOSPITAL.

SPECIAL MEETING.

A SPECIAL meeting of the governors, subscribers and other friends of the hospital was held on the 9th instant to consider what steps could be taken to free the institution from its great financial difficulties. The meeting was held in the new Clarence Memorial wing, now completed, and ready for furnishing and occupation. The situation is explained in detail in a letter which was published in our issue of the 6th instant.

The Chairman, Mr. Harbin, opened the proceedings, and the Mayor of Paddington moved the adoption of a resolution: "That this special meeting of governors and subscribers consider it imperatively necessary that the £60,000 for the completion of necessary work should be obtained without touching the small invested capital, which represents the savings of half a century, and that the annual income be increased to meet the enlarged expenditure entailed by the increased size of the hospital."

The resolution was seconded by Mr. Page, the senior surgeon to St. Mary's, and was passed unanimously.

A second important resolution was moved by the Rev. Russell Wakefield, Mayor of Marylebone, "that the meeting approves of a proposal to invite the support of residents in the districts served by the hospital by the means of local committees acting in co-operation with the central committee at the hospital."

Mr. H. P. Harris seconded the motion, which was also carried, and the meeting terminated with a vote of thanks to the chairman.

### THE HOME HOSPITALS ASSOCIATION (FOR PAYING PATIENTS), FITZROY HOUSE.

THE twenty-seventh annual general meeting of the Home Hospitals Association was held in Fitzroy Square on the 3rd inst., the Duke of Northumberland being in the chair. The minutes of the last general meeting were read and confirmed. The Report of the Committee of Management for 1904 was taken as read, every one being supplied with a printed copy. A resolution to re-elect the Duke of Northumberland as president for 1905 having been unanimously adopted, Sir Henry Burdett proposed the re-election of Mr. Frederick Cox as treasurer, which resolution was also carried unanimously.

The President then made a short speech, alluding to the important alterations and additions to the hospital carried out during the past year, and noting with satisfaction that, in spite of heavy additional expenses, the funds of the

